# Thrombosed varicocele - a rare cause for acute scrotal pain: a case report

**DOI:** 10.1186/s12894-018-0347-2

**Published:** 2018-05-08

**Authors:** M. Raghavendran, A. Venugopal, G. Kiran Kumar

**Affiliations:** 1 Department Of Urology, Apollo BGS Hospitals, Adichunchunagiri Road, Kuvempunagar, Mysore, Karnataka 570009 India; 2 Apollo BGS Hospitals, Mysore, Karnataka India; 3 Department Of Urology, Apollo Hospitals, Mysore, Karnataka India

**Keywords:** Acute Orchialgia, Thrombosed varicocele, Acute Epididymo-Orchitis, Varicocelectomy, Testicular torsion, Post Varicocelectomy thrombosis

## Abstract

**Background:**

Acute scrotal pain has various causes. Testicular torsion, torsion of appendages and Epididymo-orchitis are common causes, while varicocele thromboses are a rare cause. Varicocele thromboses can occur post operatively or spontaneously. Five cases of post-operative and five cases of spontaneous thromboses have been described till date. The traditional advice in the management of thrombosed varicocele has been to manage it conservatively in all patients by drugs and scrotal support with little description of the surgical treatment. Herein, we present an unusual sixth case of spontaneous thromboses of varicocele and discuss its presentation and surgical management. We would also like to highlight the differentiating points between spontaneous thrombosis and post operative in vitro clot formation in the varicoceles, as these two entities can often be confused for each other.

**Case presentation:**

A 68 year-old man presented with excruciating scrotal pain of one week duration. Doppler study of scrotum revealed left varicocele with no evidence of Epididymo-orchitis. He was treated with intravenous antibiotics, analgesics and scrotal elevation. He had no relief and continued to have severe pain. Clinical examination was normal. Patient underwent exploratory surgery on a semi- emergent basis. Exploration revealed normal testis with thrombosed varicoceles. Patient underwent Varicocelectomy. Postoperatively patient had immediate pain relief. Histopathology revealed prominent thrombosed varicocele. A varicocelectomy specimen (done for primary infertility) was used for comparison. The differentiating points between the two entities were noted.

**Conclusion:**

Spontaneous thrombosis of varicocele is a rare cause of acute scrotal pain. Pain out of proportion to clinical features is characteristic. Patients not responding to medical therapy may need varicocelectomy. Varicocelectomy may give immediate relief. Histopathology is useful in this disorder.

## Background

Acute scrotal pain has multiple aetiologies. Torsion of testis or its appendages and Epididymo-orchitis are common [1], while Varicocele thrombosis is a rare cause [2]. Varicocele thromboses can occur post operatively (5 cases) or spontaneously (5 cases) [2–4]. Spontaneous thrombosis can occur due to trauma or in patients with coagulation abnormalities [5]. Kayes et al. had reported that vigorous sexual or sporting activity, infections, trauma, long hour flights and drugs could cause this condition [6]. There were no major predisposing mechanisms for spontaneous thrombosis to occur in our patient, but it is possible that vigorous sexual activity could have caused this because patient developed pain after sexual intercourse. Varicocele thromboses (both spontaneous and post-operative) have been managed conservatively in all patients till date by means of drugs (antibiotics and anti-inflammatory agents) and scrotal support with no description of surgical treatment. There are contradictory reports with regards to the timing and requirement of surgery in thrombosed Varicocele patients. Roach et al. and El Hannawy et al. recommend conservative non-operative management, despite the fact that they subjected their patients to surgery. Hence the timing and requirement of surgery in thrombosed Varicocele patients becomes a hugely debatable point [7, 8]. Herein, we present a case of spontaneous Varicocele thromboses with special emphasis on its presentation and surgical management. Spontaneous in vivo thrombi has historically been mistaken for post varicocelectomy in vitro clot formations in veins. We would like to highlight the histopathological differentiation between these two distinct entities.

## Case presentation

**Fig. 1:**
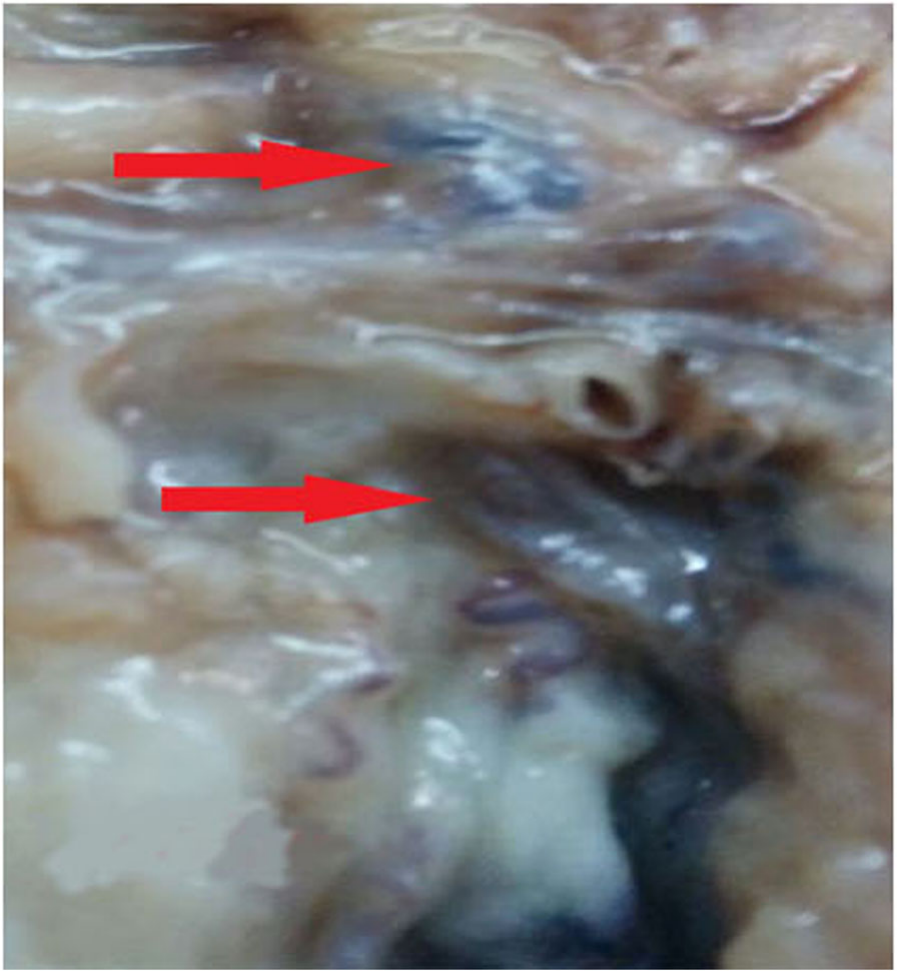
Gross photograph showing prominent blue coloured varicoceles (red arrows)

**Fig. 2:**
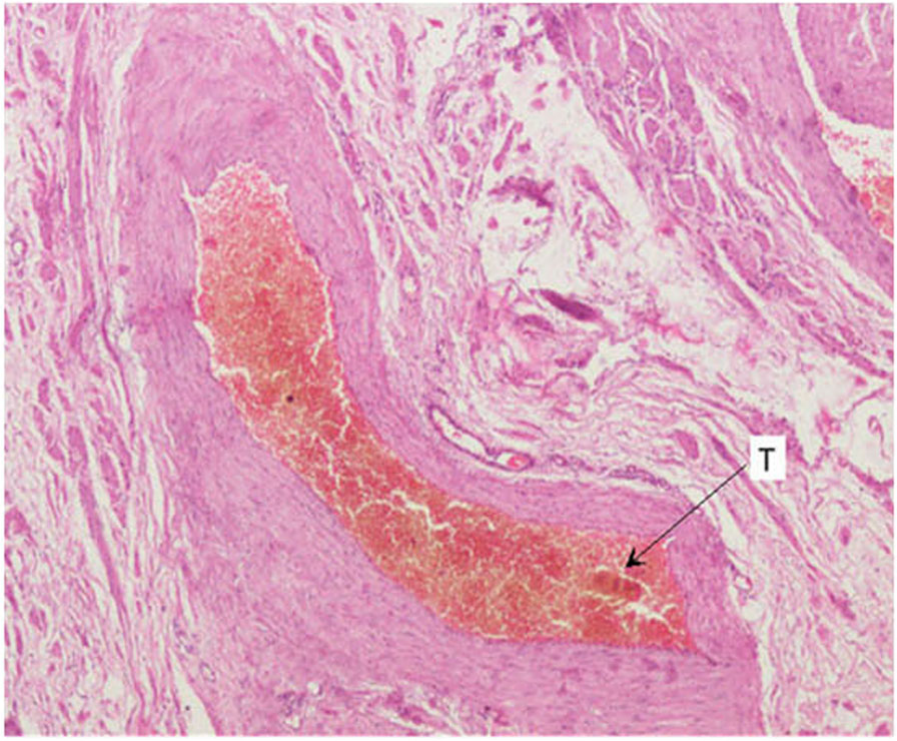
Histopathology showing prominent varicocele completely occluded by thrombus (T)

**Fig. 3:**
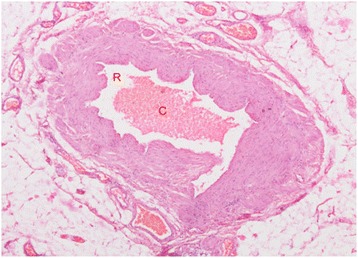
Histopathology showing a varicocele removed for primary infertility occluded by in vitro clot (C) with Retraction Space (R)

A 68 year-old man presented with excruciating left scrotal pain of one week duration. He had undergone Doppler study of the scrotum elsewhere, which revealed grade one varicocele with no evidence of Epididymo-orchitis. There was no other significant pathology in the Doppler. He was treated with intravenous antibiotics, parenteral and oral analgesics and scrotal elevation for around ten days. He had no relief and continued to have excruciating, severe pain. Pain was constant, continuous, with radiation from the scrotum to the inguinal region. Pain was not alleviated even with analgesics. There were no further aggravating factors. Clinical examination revealed normal looking scrotum with no features of inflammation. In view of pain out of proportion to clinical features, exploratory surgery was planned. Exploration revealed normal testis with blue prominent, tense and turgid varicoceles (red arrows in Fig. 1). Patient underwent left Varicocelectomy. Postoperatively patient had immediate pain relief. Histopathology showed prominent varicocele with lumen completely occluded by thrombi adherent to the wall with no retraction space (Fig. 2). A varicocelectomy specimen (done for primary infertility) was used for comparison and showed veins with in- vitro clots. The clot was not attached to the wall and there was clear retraction space between the clot and wall (Fig. 3).

## Discussion

Thrombosed varicoceles have been described as a rare cause of acute scrotal pain [2–4]. Postoperative thrombus in pampiniform plexuses have been managed conservatively with intravenous antibiotics, parenteral and oral analgesics, scrotal elevation with bed rest [3]. Spontaneously occurring thrombi have also been reported to have been managed medically in a previous case, though the exact details of medical treatment are unclear [2, 4]. As of date, all the cases mentioned in literature have been managed medically without any note of the surgical treatment needed in such cases. It is interesting to note the first report in literature by Roach et al. They had recommended conservative management. In their study of 2patients, both underwent surgical ligation and excision of the thrombosed vein [7]. So their recommendations are contrary to their findings. Similarly El Hennawy et al. advice conservative management, when in their case they subjected the patient to surgery [8]. There are 2 other case reports which have reported that pain usually subsides with a week of non-steroidal anti-inflammatory medications and scrotal rest. Here also, Doerfler et al. recommend medical management, while their patient was subjected to surgery [2]. Kleinclauss et al. were the only ones who managed their patient successfully with medical management alone [4]. The other documentation of medical management is in that of post-operative Varicocelectomy patients. Summation of all the reports make us come to a conclusion that medical therapy may be successful if only a single superficial spermatic vein is involved, while in cases like ours where the majority of pampiniform plexus is thrombosed, surgical management will have a better outcome. This conclusion is similar to the one by Bolat et al. who opined that treatment can be started conservatively, with surgical intervention reserved for failed cases on an emergent basis [5]. Similarly Isenberg et al. advice that though venography, Doppler can be diagnostic, surgery should not be delayed in doubtful cases [9]. Hence, we feel that if severe pain persists in spite of adequate medical therapy (non-steroidal anti-inflammatory agents, scrotal elevation and rest for 7-10 days), as seen in our case, then these patients should be subjected to immediate surgical exploration. Varicocelectomy gives complete pain relief and should be considered as treatment of choice in this sub group of patients, who have failed medical management. Another controversial issue is whether to perform just ligation of the vein or to completely excise the segment of the thrombosed vein? Mallat et al. also reported a case where they had done complete excision of the thrombosed vein [10]. We also did surgical excision of the thrombosed vein as we felt that doing a simple ligation may not alleviate the pain completely. Another worrisome consideration is that delay in performing Varicocelectomy may probably lead to ischemic damage to the testis. Roach et al. had to perform orchiectomy due to severe venous congestion and testicular ischemia in one of their patients [7]. Hence, we postulate that in patients with severe scrotal pain not subsiding with 7 days of medical therapy, exploration and varicocelectomy should be immediately considered and may result in salvage of the testis. The second issue in these patients are do thrombi occur in varicoceles? As seen in our case, it is pretty clear that spontaneous thrombosis do occur in varicoceles. The histopathology can help in differentiating an in vivo thrombus from an in vitro post operative clot. A long standing clot in Varicocelectomy specimen of infertility will remodel and have a retraction space, while the clot in thrombosed specimen may not have this space due to the acuteness of the episode.

### Learning points

1. Spontaneous thrombosis of varicocele is a rare cause of acute scrotal pain.
2. Pain out of proportion to clinical features is characteristic of this condition.
3. Spontaneous thrombus in varicoceles not responding to adequate (7-10) medical therapy need varicocelectomy. Varicocelectomy produces immediate pain relief.
4. Histopathology can be useful in this disorder as it can help in differentiating a spontaneous complete thrombus in the acute thrombosed varicocele from an in vitro remodelled clot in a longer standing Varicocele.

## Conclusion

Spontaneous thrombosis of varicocele is a rare cause of acute scrotal pain. Pain out of proportion to clinical features is characteristic. Patients not responding to medical therapy need varicocelectomy. Varicocelectomy gives immediate relief. Histopathology is useful in this disorder.
